# Depletion of growth differentiation factor 15 (GDF15) leads to mitochondrial dysfunction and premature senescence in human dermal fibroblasts

**DOI:** 10.1111/acel.13752

**Published:** 2022-12-22

**Authors:** Sophia Wedel, Ines Martic, Lena Guerrero Navarro, Christian Ploner, Gerhard Pierer, Pidder Jansen‐Dürr, Maria Cavinato

**Affiliations:** ^1^ Institute for Biochemical Aging Research University of Innsbruck Innsbruck Austria; ^2^ Center for Molecular Biosciences Innsbruck (CMBI) Innsbruck Austria; ^3^ Department of Plastic, Reconstructive and Aesthetic Surgery Medical University of Innsbruck Innsbruck Austria

**Keywords:** GDF15, lipofuscin, mitochondria, mitokine, senescence, skin aging

## Abstract

Growth differentiation factor 15 (GDF15) is a stress‐responsive cytokine also known as a mitokine; however, its role in mitochondrial homeostasis and cellular senescence remained elusive. We show here that knocking down GDF15 expression in human dermal fibroblasts induced mitochondrial dysfunction and premature senescence, associated with a distinct senescence‐associated secretory phenotype. Fibroblast‐specific loss of GDF15 expression in a model of 3D reconstructed human skin induced epidermal thinning, a hallmark of skin aging. Our results suggest GDF15 to play a so far undisclosed role in mitochondrial homeostasis to delay both the onset of cellular senescence and the appearance of age‐related changes in a 3D human skin model.

## Graphical abstract

Depletion of growth differentiation factor 15 (GDF15) leads to mitochondrial dysfunction and premature senescence in human dermal fibroblasts. GDF15, a mitokine regulated in response to different sources of stress, is upregulated during cellular senescence and in the plasma of aged animals, including humans. In normal cells, under stress conditions, transcription and secretion of GDF15 are increased, although the mechanisms of intra and extracellular action of this protein are still not fully understood. Here, we demonstrate that knockdown of GDF15 induces premature senescence in human dermal fibroblasts. In this context, senescence was characterized by the accumulation of ROS, mitochondrial dysfunction, and secretion of a distinct SASP. When inserted in 3D skin equivalents, senescent GDF15 knockdown fibroblasts induced epidermal thinning, a common characteristic of skin aging, supporting the important role of GDF15 in maintenance of cell homeostasis.

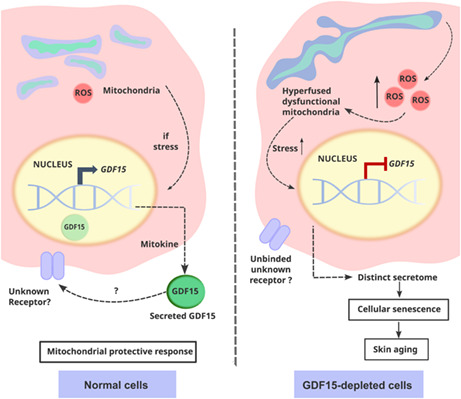

GDF15 expression is upregulated in response to a wide range of different cellular stressors and is accepted as a biomarker for aging and senescence (Cardoso et al., 2018). To investigate the function of GDF15 in senescence induction, human foreskin fibroblasts (HFF), used as a convenient model to study the development of senescence in cells from newborn humans and reflecting the most juvenile phenotype available, were transduced with lentiviral particles carrying either shRNA targeting GDF15 gene expression (GDF15KD) or scrambled shRNA (control) and cultured for 12 weeks. Cells were categorized into early passage (EP, passage 8–13), early‐middle passage (EMP, passage 16–19), late‐middle passage (LMP, passage 23–24), and late passage (LP, passage 27–28) (Figure 1a). As expected, GDF15 levels increased gradually with passage number in control cells, whereas both GDF15 mRNA level and protein secretion were reduced in GDF15KD cells (Figure 1b,c). GDF15KD cells underwent significantly less cumulative population doublings (cPDL) compared with controls after 20 days in culture (Figure 1a). GDF15KD cells displayed increased cell surface area (Figure 1d) and increased senescence‐associated β‐galactosidase (SA‐β‐Gal) activity (Figure 1e,f), along with additional senescence markers (Figure S1A–E), including lipofuscin (Figure 1g, Figure S1F; von Zglinicki et al., 1995).

**FIGURE 1:**
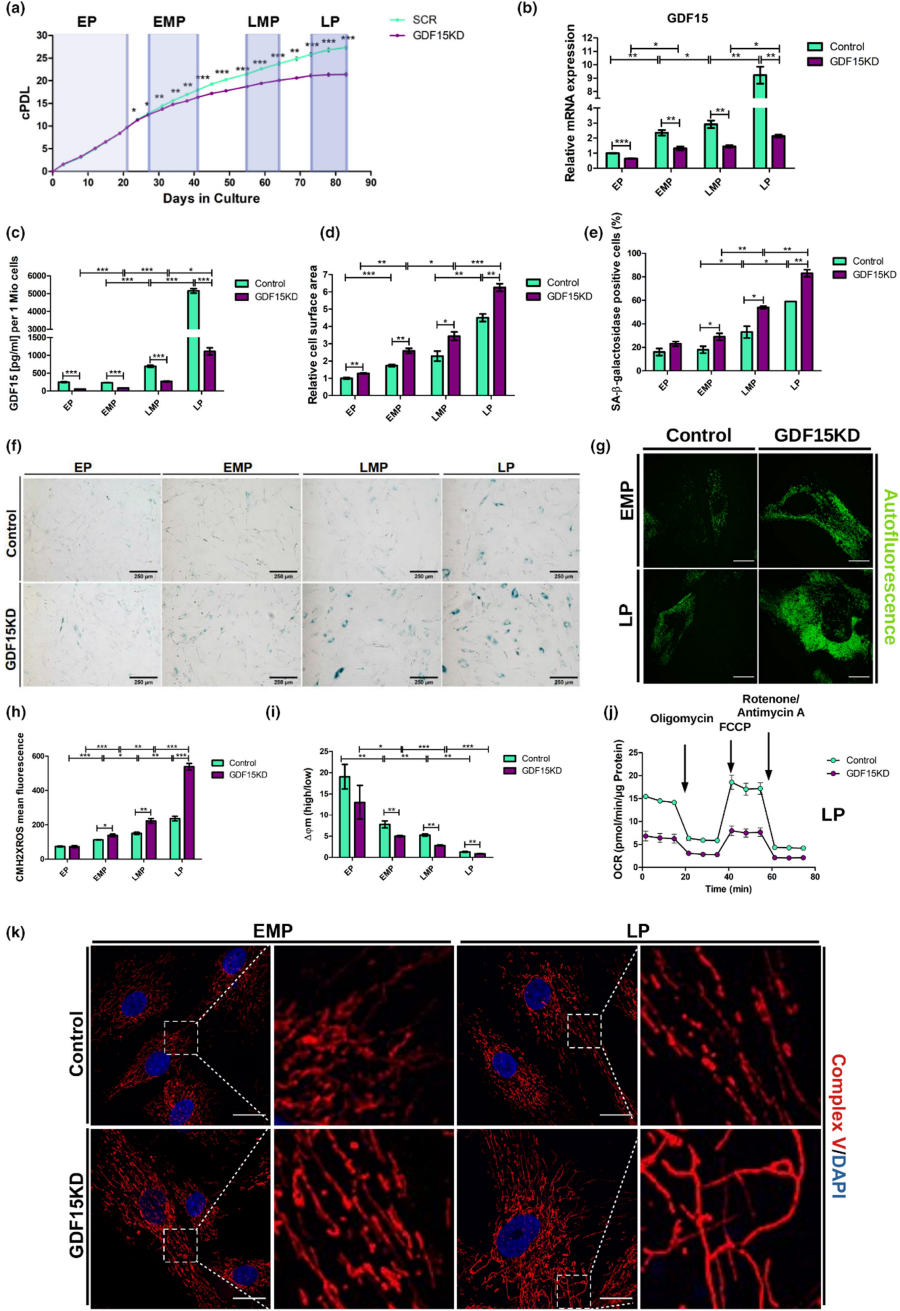
GDF15 knockdown in HFF induces premature senescence associated with mitochondrial dysfunction. HFF were transduced with lentiviral vectors carrying GDF15 shRNA or control shRNA and grown under selection. (a) GDF15KD and control cells were cultured over an extended period of time, and cPDL were calculated. Defined measurement intervals that summarize cells across certain passages are indicated as EP = early passage, EMP = early‐middle passage, LMP = late‐middle passage, LP = late passage; (b) relative GDF15 mRNA levels were estimated by qRT‐PCR. (c) Secretion of GDF15 protein was measured using ELISA. For each time point, (d) relative cell surface area and (e) percentage of SA‐β‐Gal‐positive cells were determined. (f) Representative pictures of SA‐β‐Gal staining are shown. (g) Autofluorescence of GDF15KD and control cells was recorded by confocal live‐cell imaging. Representative pictures are shown. Scale bar = 25 μm; CM‐H2XRos (h) and JC‐1 (i) staining were used to measure mitochondrial ROS and mitochondrial membrane potential using flow cytometry, respectively. Data present mean values ± SD, *n* = 3; (J) OCR of GDF15KD and control cells were measured using Seahorse Flux Analyzer after the successive injection of oligomycin, FCCP, and a mixture of antimycin A and rotenone, as indicated. Data present mean values ± , *n* = 4; (k) cells were processed for IF using a primary antibody against Complex V. Representative pictures are shown. Scale bar = 25 μm; statistical analysis was calculated using *t*‐test (*p < 0.05, **p < 0.01, ***p < 0.001)

GDF15 is upregulated upon mitochondrial dysfunction (Fujita et al., 2016) and is thought to function as a mitokine (Conte et al., 2019), yet its potential to restrict mitochondrial damage has not been directly demonstrated. GDF15KD cells showed increased mitochondrial ROS (Figure 1h) and decreased mitochondrial membrane potential (Figure 1i, Figure S2A) compared with controls, implying that mitochondrial damage accumulated over time. No significant differences were observed when comparing mitochondrial DNA content between control and GDF15KD cells (Figure S2B). Assessment of oxygen consumption rates (OCR) revealed a significant reduction of both basal and ATP‐coupled respiration in GDF15KD cells compared with controls, which was accompanied by significantly decreased respiratory capacity (Figure 1j, Figure S2C‐D). Moreover, we examined mitochondrial network integrity by indirect immunofluorescence (IF) staining to detect ATPase β, a Complex V subunit, which revealed the accumulation of increasingly elongated mitochondria in GDF15KD cells relative to control (Figure 1k, Figure S2E). Mitochondrial network dynamics becomes progressively inflexible with senescence, due to decreased frequencies of mitochondrial fission and fusion events (Dalle Pezze et al., 2014; Mai et al., 2010). We consider either reduced rate of mitochondrial fission events or hyperfusion to account for the premature elongation of mitochondria in GDF15KD cells. The accumulation of elongated, yet damaged mitochondria has been previously linked to insufficient autophagy (Tang et al., 2019; Towers et al., 2021). While these findings would suggest a possible connection between GDF15 and autophagy, the role of GDF15 in regulating mitochondrial network dynamics and autophagy remains to be elucidated.

Major age‐related skin alterations are caused by the increased expression and secretion of cytokines and matrix metalloproteinases (MMPs) by both keratinocytes and fibroblasts (Fisher et al., 2002; Kim & Park, 2016; Sárdy, 2009). To investigate the secretome of HFFs, we analyzed the expression pattern of various SASP components of control and GDF15KD HFF. Using qRT‐PCR, we found IL1a as well as several MMPs, including MMP3, MMP10, and MMP12, strongly upregulated in GDF15KD HFF on mRNA level (Figure 2 A‐DD‐F). Increased expression of MMPs was further confirmed on protein level by MMP array using supernatant from cells in LP (Figure 2e). Our data suggest that senescence of HFF induced by GDF15KD is accompanied by mitochondrial dysfunction and correlated with a distinct secretion profile.

**FIGURE 2:**
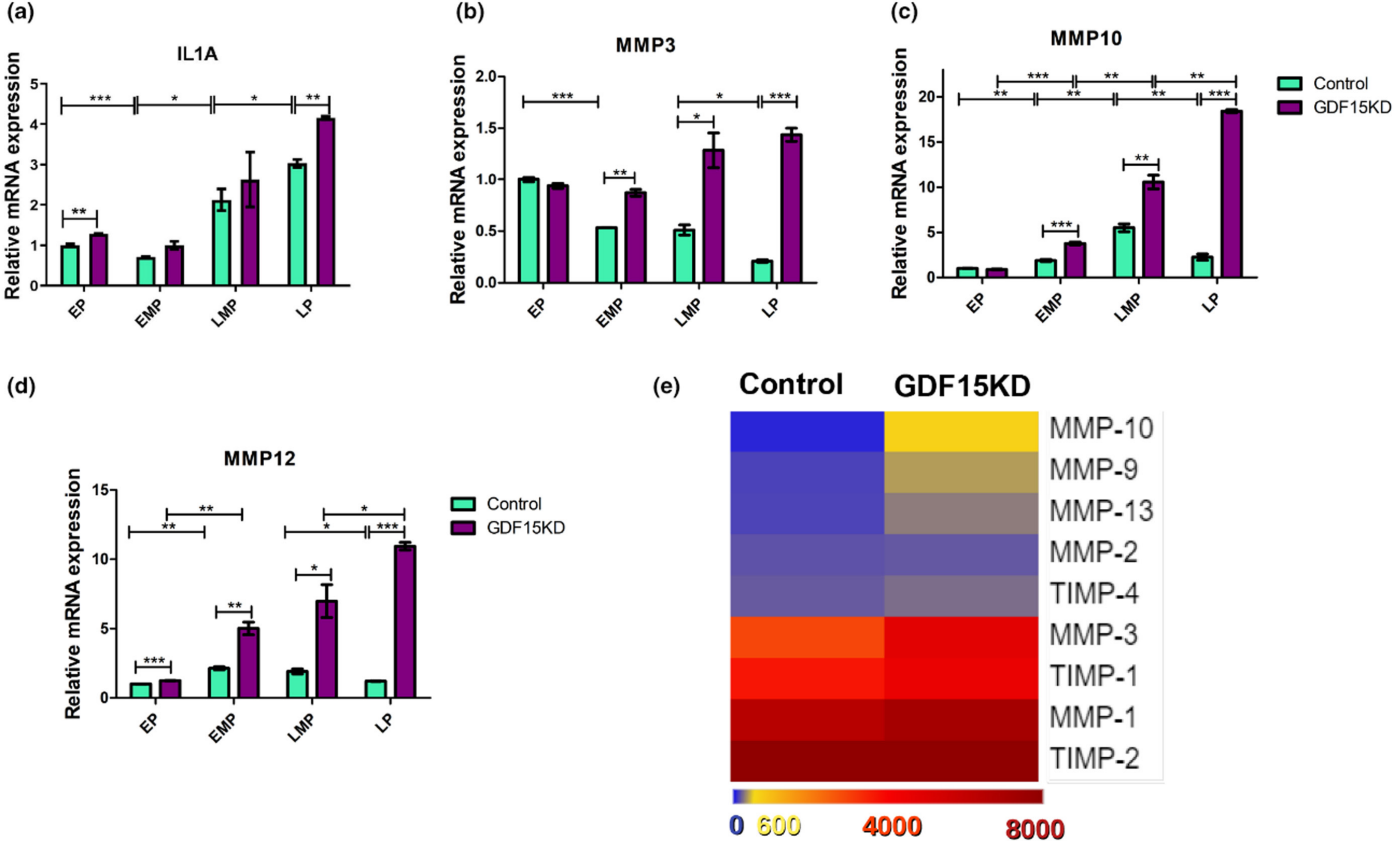
GDF15 knockdown induces a distinct SASP in HFF. (a–d) Relative mRNA levels were estimated by qRT‐PCR in control and GDF15KD HFF for (a) IL1a and several MMPs, including (b) MMP3, (c) MMP10, and (d) MMP12. Data present mean ± SD, *n* = 3; (e) MMP arrays were performed with supernatant from GDF15KD, and control HFF and data were summarized in a heatmap. Data present mean values, *n* = 4; statistical analysis was calculated using *t*‐test (*p < 0.05, **p < 0.01, ***p < 0.001)

Recent evidence suggests that skin homeostasis depends on elaborate intratissue communication between the dermal and epidermal compartments, which seems to be perturbed during skin aging (Kim et al., 2020; Weinmüllner et al., 2020; Yoon et al., 2018). The appearance and persistence of senescent cells in the skin has been reported to contribute to age‐related changes and pathologies (Wang & Dreesen, 2018). Moreover, the presence of senescent cells in a certain layer of the skin can influence the aging process of the tissue as a whole (Victorelli et al., 2019). To investigate a potential role of GDF15 in skin aging, dermal fibroblasts (HSDF) and epidermal keratinocytes (HSEK) were isolated from the skin of healthy donors. Subsequently, GDF15 expression was knocked down in HSDF by lentiviral shRNA transduction, which, as expected, increased the percentage of SA‐ß‐Gal‐positive cells (Figure 3a). GDF15KD HSDF and nontransduced HSEK from the same donor were then used for the production of 3D skin equivalents (SE). SE produced with nontransduced HSDF and HSEK from the same donor served as control. Hematoxylin and eosin (H&E) staining of the tissue revealed significant structural changes, as skin equivalents composed of GDF15KD HSDF showed decreased epidermal thickness compared with SE produced with control HSDF (Figure 3b,c). These results indicated that the integration of GDF15KD fibroblasts in SE triggered epidermal thinning, a hallmark of skin aging. This may be due to GDF15‐dependent changes in the cellular secretome, possibly including tissue‐deteriorating factors, such as matrix metalloproteases (MMPs), which are well known to drive tissue structure remodeling during skin aging (Kim & Park, 2016). To investigate the possibility of senescence‐associated secretory phenotype (SASP) factors being responsible for the observed structural changes in GDF15‐deficient SE, we analyzed the expression pattern of various SASP components. In contrast to what we observed in HFF (Figure 2), GDF15KD HSDF did not show increased expression of MMP3, MMP10, and MMP12 in comparison with their control counterparts (Figure S3A‐C), which could be partially explained by the already elevated levels of these MMPs expressed by HSDF when compared to HFF (Figure S3D‐F). Instead, the secretome of HSDF GDF15KD was mainly characterized by increased expression of MMP1 and IL6 (Figure 3d,e). Taken together, these results suggest that GDF15 deficiency in HSDF induced the upregulation of different SASP members, driving alterations in extracellular matrix composition in the dermis. Inclusion of senescent fibroblasts in SE has been described to cause epidermal thinning in a model of human skin aging (Weinmüllner et al., 2020), reflecting an elaborate interaction between the dermal and epidermal compartment during skin aging (Kim et al., 2020; Yoon et al., 2018).

**FIGURE 3:**
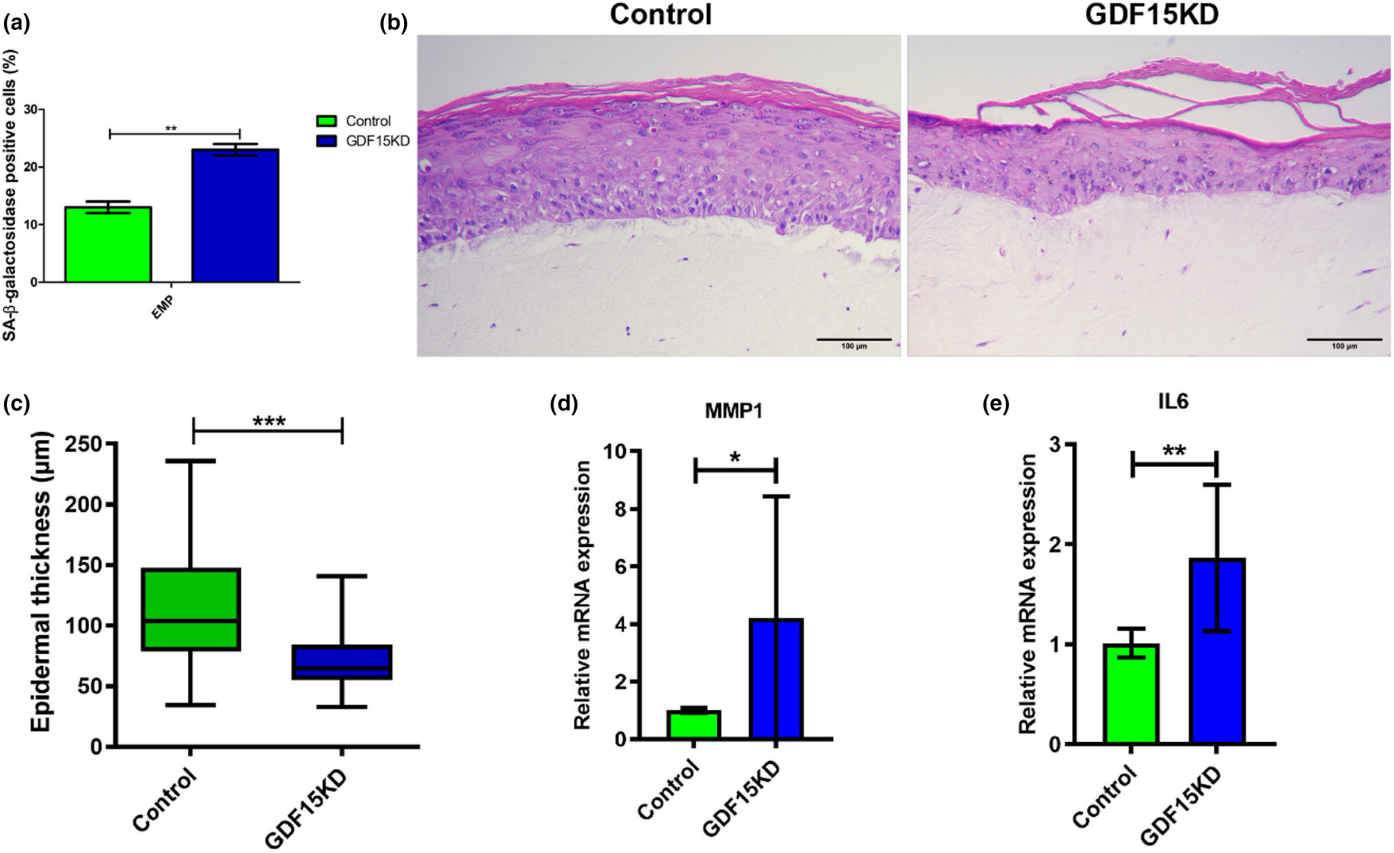
GDF15 knockdown changes SASP composition of HSDF and reduces epidermal thickness in 3D skin equivalents. HSDF were transduced with lentiviral vectors carrying GDF15 shRNA or control shRNA and grown under selection. (a) The percentage of SA‐β‐Gal‐positive cells was estimated. (b) Skin equivalents containing either GDF15KD or control HSDF were stained with H&E. (c) Epidermal thickness [μm] was measured using ImageJ software. Data present mean values ± SD, *n* = 4; 50 to 55 epidermal sites (excluding *stratum corneum*) were measured per skin equivalent. *N* = 9 (3 different donors); (c–e) relative mRNA levels were estimated by qRT‐PCR in control and GDF15KD HFF for MMP1 (d) and IL‐6 (e). Data present mean values ± SD, *n* = 3; statistical analysis was calculated using *t*‐test (*p < 0.05, **p < 0.01, ***p < 0.001)

To investigate the potential mechanism of action of GDF15 in the skin extracellular space, we determined the expression GFRAL by qRT‐PCR in lysates of keratinocytes and fibroblasts (Table S2). Although GFRAL was described to be the GDF15 receptor in the central nervous system and in adipose tissue (Conte et al., 2022; Yang et al., 2017), we found very low to undetectable levels of GFRAL mRNA in skin keratinocytes and fibroblasts, suggesting that in skin cells GDF15 might primarily act intracellularly or interact with cells in the extracellular space via an alternative receptor.

In short, our results demonstrate that GDF15 deficiency induces premature senescence in HFF that is characterized by the accumulation of dysfunctional mitochondria. GDF15KD fibroblasts express a distinct SASP that affects adjacent keratinocytes and leads to epidermal thinning in 3D skin equivalents.

## AUTHOR CONTRIBUTIONS

S.W., P.J.D., and M.C. designed the study. C.P. and G.P. contributed to experimental design. S.W., I.M., and L.G.N. performed the experiments and analyzed the data. S.W., P.J.D., and M.C. wrote the manuscript with input from I.M., L.G.N., C.P., and G.P.

## AKNOWLEDGEMENTS

We thank Annabella Pittl for her valuable contribution to this study.

## FUNDING INFORMATION

This work was supported by a scholarship of the University of Innsbruck (Doktoratsstipendium aus der Nachwuchsförderung) granted to S.W. and I.M., a grant from the Austrian Science Funds (FWF, grant # P 31582) to P.J.D. and by the European Commission (COFUND‐ARDRE, Project # 847681) to P.J.D. and L.G.N.

## CONFLICTS OF INTEREST

None.

## Supporting information


Appendix S1

## Data Availability

The data are available from the corresponding author upon reasonable request.
