# Supplementary material for: Depletion of growth differentiation factor 15 (GDF15) leads to mitochondrial dysfunction and premature senescence in human dermal fibroblasts
Source: Aging Cell. 2022 Dec 22;22(1):e13752. doi: 10.1111/acel.13752 (PMC9835581; doi:10.1111/acel.13752)
Supplement: Supplementary file 1 — Appendix S1 [file ACEL-22-e13752-s001.docx]

**Supplementary Material**

**Extended Methods**

**Chemicals:**

All chemicals were purchased from Sigma (Steinheim, Germany) unless stated otherwise.

**Cell Culture, Estimation of Cumulative Population Doublings (cPDL) and Cell Surface Area**

Human diploid fibroblasts derived from newborn foreskin (HFF-1, ATCC® #SCRC-1041, Manassas, Virginia, USA) and human skin dermal fibroblasts (HSDF) were cultivated in Dulbecco’s Modified Eagle’s Medium (DMEM D5546, Sigma, Steinheim Germany) with 10% fetal bovine serum, 4 mM L-Glutamine and 1% Penicillin-Streptomycin. Human skin epidermal keratinocytes (HSEK) were cultivated in Dermalife K Medium (LL-000, Lifeline Cell Technology, USA). HSEK and HSDF were isolated from abdominal skin after plastic surgery as described (Fernandez et al., 2014). The skin was donated by patients from the Department of Plastic, Reconstructive and Aesthetic Surgery, Medical University of Innsbruck, with informed consent according to current guidelines of the ethic committee, Medical University Innsbruck. Cells were counted, cPDL were estimated and cell surface area was measured as described (Wedel et al., 2020).

**Stable Knockdown of GDF15 expression**

Plasmid DNA from SMARTvector™ lentiviral shRNA glycerol stocks (Dharmacon, USA) was isolated after expanding bacterial culture using the Qiagen® Plasmid Maxi Kit (#12165, Qiagen) according to the manufacturer’s protocol. Lentiviral particles were produced as described by Greussing et al. (2013). The viral supernatant was used to infect HFF-1 and HSDF, which were continuously cultured under puromycin selection starting two days after infection. A lentiviral vector expressing a scrambled shRNA sequence was used as control.

**GDF15 ELISA**

Supernatant of fibroblasts was collected 24h - 48h after media change and concentrated using AMICON Ultra-15 tubes (Merck Millipore, USA). To detect and measure GDF15 concentration in cellular supernatant the Human Quantikine ELISA Kit (#DGD150, R&D Systems, USA) was used according to manufacturer’s protocol.

**RNA Isolation, cDNA Synthesis and RT-qPCR**

RNA isolation, cDNA synthesis and RT-qPCR were performed as described (Wedel et al., 2020). See Supplementary Table 1 for primer sequences used.

**Protein Isolation and Westernblot**

Protein lysates were obtained as previously described by Wedel et al. (2020). Protein content was separated using SDS-PAGE. Transfer to PVDF membranes was performed according to a standard protocol as described by Greussing et al. (2013). The following primary antibodies were used: anti-Lamin B1 (#ab16048, Abcam, United Kingdom), anti-phospho-pRB (#9308S Cell signaling technology, USA) and anti-GAPDH (#SC-25778, Santa Cruz Biotechnology, USA). Chemiluminescence substrate kit (Merck Millipore, USA) and ChemiDoc Imaging System (Biorad Laboratories, USA) were used for detection.

**Cytochemistry for Senescence-Associated-β-galactosidase (SA-β-Gal)**

SA-β-galactosidase staining was performed as described (Greussing et al., 2013). A minimum of 400 cells per sample were counted and the percentage of SA-β-galactosidase positive cells was calculated by dividing the number of blue cells by the total number of cells counted.

**Immunofluorescence**

Immunofluorescence was performed according to standard protocol (Wedel et al., 2020). Following primary antibody was used: anti-ATP Synthase beta (#A21351, Thermo Fisher, Austria).

**Measurement of oxygen consumption rates using Seahorse technology**

Oxygen consumption rates were analyzed using the Seahorse XF HS Mini (Agilent Technologies, USA). 8000 to 10000 cells were seeded one day before the assay into Seahorse HS Miniplates. To assess mitochondrial function, the OCR was measured with Seahorse Cell Mito Stress Test protocol, including 1µM oligomycin, 2µM FCCP and 0.5µM Antimycin A/Rotenone mix injection. Oxygen consumption rates were normalized to protein concentration as described by the manufacturer.

**MMP Arrays**

Human MMP Antibody Arrays (#ab134004, Abcam, United Kingdom) were probed with cellular supernatant and developed according to manufacturer’s protocol.

**Relative mitochondrial DNA (mtDNA) / nuclear DNA (nDNA) ratio**

Relative mtDNA and genomic/nuclear DNA were assessed by qPCR using appropriate primers (Table S1) as described (Wedel et al., 2020).

**Live Cell Imaging**

To detect lipofuscin-associated autofluorescence, the cells were seeded into glass bottom dishes (Greiner Bio-One, Austria) and imaged using a Cell Voyager CV1000 Yokogawa (Visitron Systems, Germany). Evaluation of fluorescence intensity was performed using ImageJ software.

**Production and Processing for Histology of 3D Reconstructed Skin (Skin Equivalents)**

For the production of 3D skin equivalents WT and GDF15KD HSDF and patient matched HSEK from 3 different donors (age range 37-57y) were used. 3D skin equivalents were produced as described (Cavinato et al., 2016). In brief, HSDF were seeded in collagen matrix in deep well plates and allowed to polymerize to form the dermal equivalent. On the next day, keratinocytes were seeded on top of the dermal equivalent and the full construct was maintained submerged in keratinocytes’ growth medium overnight (DermaLife K - keratinocytes medium, Life Line cell technology, Maryland, USA). Next, the skin equivalents were submitted to air-liquid interface (ALI) and the medium was replaced by keratinocytes differentiation medium (KGM Bullet Kit, Lonza, supplemented with 25 mg of ascorbic acid, 10 µg/mL of transferrin, 500mg BSA, and 1 M of CaCl_2_). Seven days after the beginning of the ALI 3D skin equivalents were fixed, processed, stained and analyzed as described (Wedel et al., 2020).

**Statistical analysis**

If not differently stated, results are displayed as mean values ± standard deviation (SD) of n=3. Statistical analysis was done using Student’s t-test.

**References**

Cavinato, M., Koziel, R., Romani, N., Weinmüllner, R., Jenewein, B., Hermann, M., Dubrac, S., Ratzinger, G., Grillari, J., Schmuth, M., Jansen-Dürr, P., 2016. UVB-Induced Senescence of Human Dermal Fibroblasts Involves Impairment of Proteasome and Enhanced Autophagic Activity. Journals Gerontol. - Ser. A Biol. Sci. Med. Sci. 72, glw150. https://doi.org/10.1093/gerona/glw150

Fernandez, T.L., Van Lonkhuyzen, D.R., Dawson, R.A., Kimlin, M.G., Upton, Z., 2014. Characterization of a human skin equivalent model to study the effects of ultraviolet B radiation on keratinocytes. Tissue Eng. Part C. Methods 20, 588–98. https://doi.org/10.1089/ten.TEC.2013.0293

Greussing, R., Hackl, M., Charoentong, P., Pauck, A., Monteforte, R., Cavinato, M., Hofer, E., Scheideler, M., Neuhaus, M., Micutkova, L., Mueck, C., Trajanoski, Z., Grillari, J., Jansen-Dürr, P., 2013. Identification of microRNA-mRNA functional interactions in UVB-induced senescence of human diploid fibroblasts. BMC Genomics 14, 224. https://doi.org/10.1186/1471-2164-14-224

Wedel, S., Martic, I., Hrapovic, N., Fabre, S., Madreiter-Sokolowski, C.T., Haller, T., Pierer, G., Ploner, C., Jansen-Dürr, P., Cavinato, M., 2020. tBHP treatment as a model for cellular senescence and pollution-induced skin aging. Mech. Ageing Dev. 190, 111318. https://doi.org/10.1016/j.mad.2020.111318

**Supplementary Table 1:** Target gene specific primer pair sequences used for qRT-PCR.

| Gene | Sequences |
| --- | --- |
| p16 | GAT TGA AAG AAC CAG AGA GGC |
|  | TGA AAA CTA CGA AAG CGG G |
| GAPDH | GAG TCA ACG GAT TTG GTC GT |
|  | GAT CTC GCT CCT GGA AGA TG |
| MMP3 | TGC TGC TCA TGA AAT TGG CC |
|  | TCA TCT TGA GAC AGG CGG AA |
| MMP10 | GAC AGA AGA TGC ATC AGG CAC |
|  | GGC GAG CTC TGT GAA TGA GT |
| MMP12 | ACA CAT TCA GGA GGC ACA AAC |
|  | ATG TCA TCA GCA GAG AGG CG |
| LAMINB1 | AAG CAG CTG GAG TGG TTG TT |
|  | TTG GAT GCT CTT GGG GTT C |
| GFRAL | GCC TCA GTC TAA TTC GCA G |
|  | TTA GAC AGG GTT GGA TAA TTG |
| MMP1 | CAT CGT GTT GCA GCT CAT GA |
|  | ATG GGC TGG ACA GGA TTT TG |
| IL1a | TCA GCA AAG AAG TCA AGA TGG C |
|  | CAT GGA GTG GGC CAT AGC TT |
| IL6 | AAG CCA GAG CTG TGC AGA TGA GTA |
|  | TGT CCT GCA GCC ACT GGT TC |
| MT-ND4 | ACT CTC ACT GCC CAA GAA CT |
|  | TGA GGC GTA TTA TAC CA |
| MT-COX1 | TAC GTT GTA GCC CAC TTC CAC T |
|  | AGT AAC GTC GGG GCA TTC CG |

**Supplementary Table 2:** CT values resulting from q-RT-PCR to detect the expression of GFRAL mRNA in HSDF and HSEK. n.d: non-detectable (above 40 cycles)

|  | CT | | | | | |
| --- | --- | --- | --- | --- | --- | --- |
| HSDF 1 WT | 36,874 | n.d. | n.d. | n.d. | n.d. | n.d. |
| HSDF 2 WT | n.d. | n.d. | 34,899 | 35,581 | 37,890 | n.d. |
| HSDF 3 WT | n.d. | 35,277 | 35,658 | 35,050 | n.d. | n.d. |
| HSDF 1 GDF15KD | n.d. | n.d. | n.d. | n.d. | n.d. | n.d. |
| HSDF 2 GDF15KD | n.d. | 37,518 | 36,565 | 36,988 | 34,340 | n.d. |
| HSDF 3 GDF15KD | n.d. | n.d. | 38,585 | 36,671 | 35,414 | 36,254 |
| HSEK | n.d. | n.d. | n.d. |  |  |  |

**
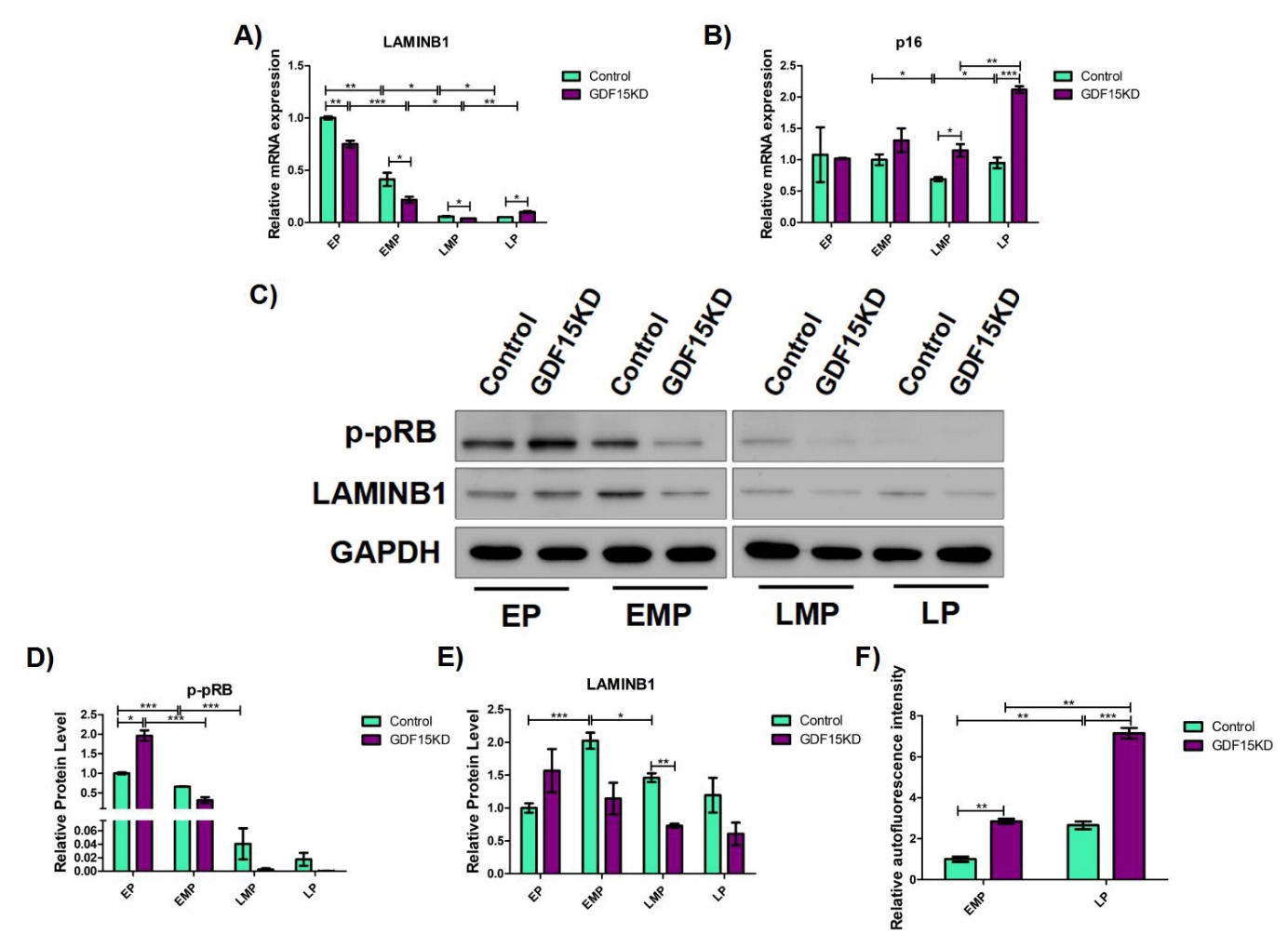
**

**Figure S1** A-B) The expression of senescence markers, including (A) LAMINB1 and (B) p16, was analyzed by qRT-PCR. C) p-pRB and LAMINB1 protein levels were analyzed by immuoblotting. D) p-pRB and (E) LAMINB1 protein levels were quantified. Data presents mean values ± SD, n=3; F) Autofluorescence intensity of GDF15KD and control cells in EMP and LP was recorded by confocal live-cell imaging and subsequent analysis using ImageJ software. 30 cells per sample were measured and three independent samples were analyzed by group. Statistical analysis was calculated using t-test (*P < 0.05, **P < 0.01, ***P < 0.001)


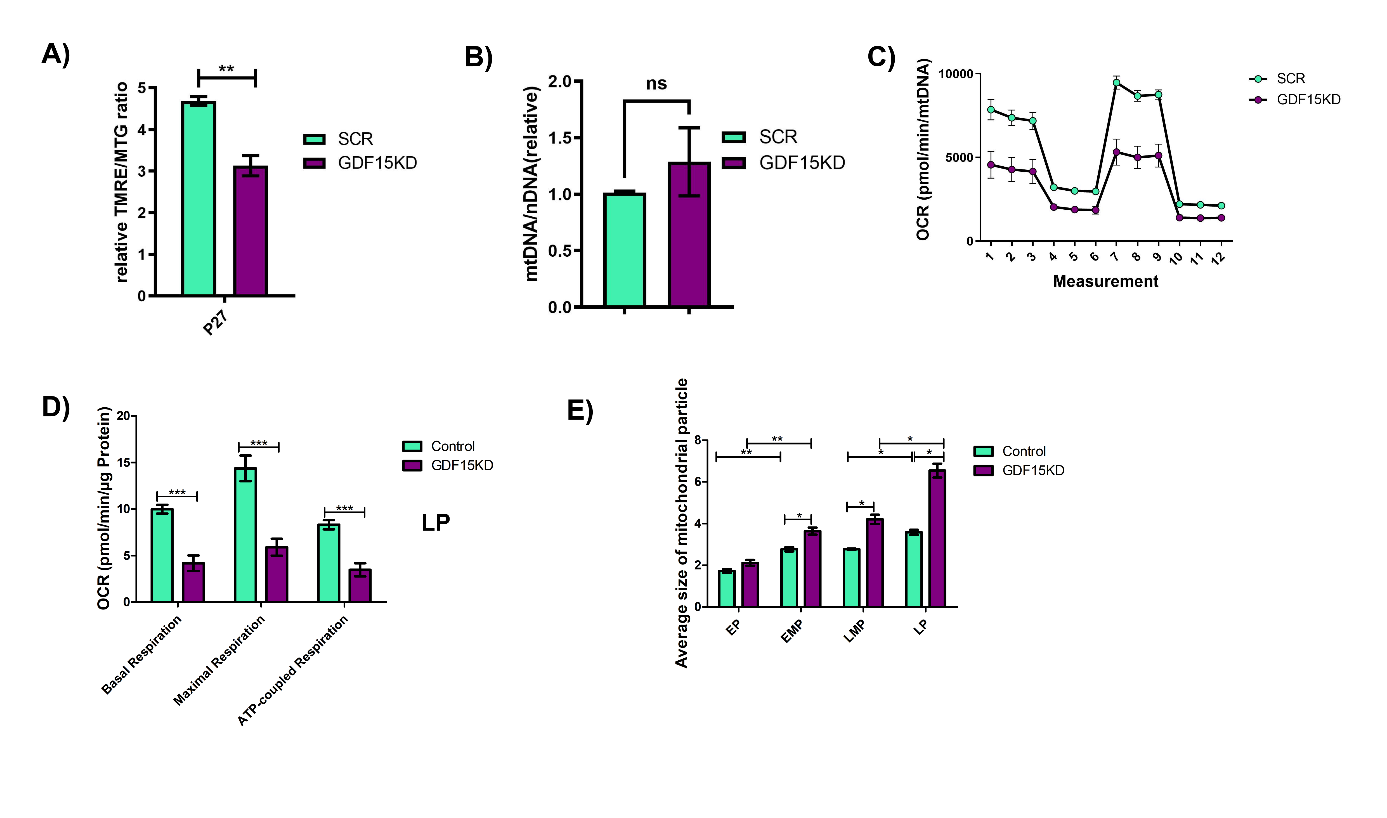


**Figure S2** A) Changes in mitochondrial membrane potential in response to GDF15KD in HFF were assessed by FACS using the fluorescent probe TMRM. Total mitochondrial mass was assessed by the same means using Mitotracker Green (MTG). Changes in mitochondrial membrane potential are presented as TMRM mean fluorescence normalized to MTG. B) mtDNA/nDNA ratio was measured by qPCR; Data represents mean values ± SD. C) OCR of GDF15KD and control cells in LP were measured using Seahorse Flux Analyzer after the successive injection of oligomycin, FCCP, and a mixture of antimycin A and rotenone and values were normalized to mtDNA content. D) Mitochondrial respiration of control and GDF15KD cells in LP was measured using Seahorse Flux Analyzer. Respiratory parameters including basal, maximal and ATP-coupled respiration were calculated for control and GDF15KD cells after normalization to protein content. Data presents mean values ± SD; n=4; E) Average size of mitochondrial particles based on IF was analyzed using ImageJ software. Per sample 50 cells were measured, n=3; Statistical analysis was calculated using t-test (*P < 0.05, **P < 0.01, ***P < 0.001).


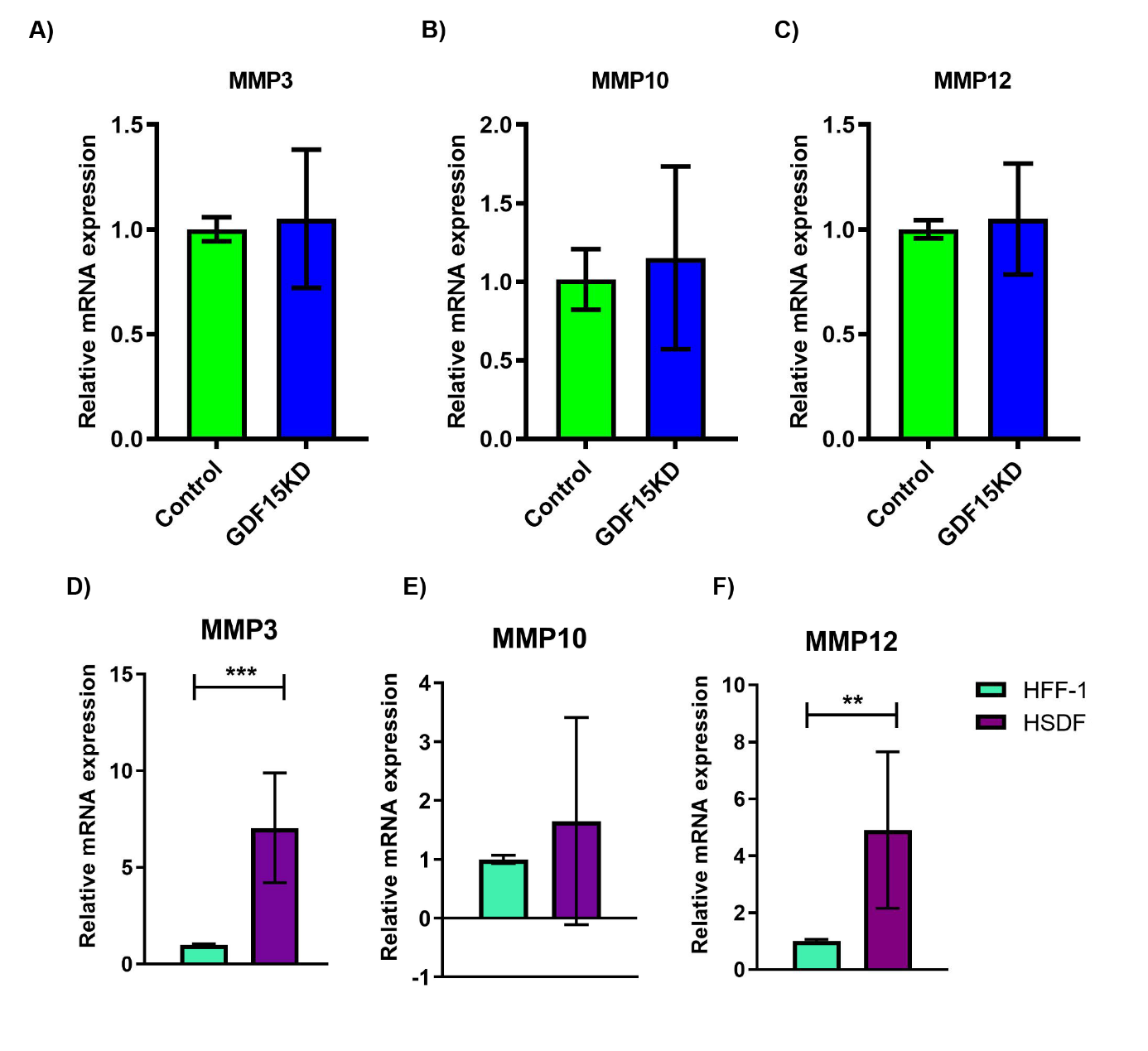


**Figure S3:** (A-C) Relative mRNA levels were estimated by q-RT-PCR in control and GDF15KD HSDF for (A) MMP3, (B) MMP10 and (C) MMP12. Data presents mean values ± SD, n=3; (D-F). Comparison of mRNA levels of (D) MMP3, (E) MMP10 and (F) MMP12 assessed by q-RT-PCR in HFF and HSDF. Data presents mean values, n=4; Statistical analysis was calculated using t-test (*P < 0.05, **P < 0.01, ***P < 0.001)
